# A Case of Severe Lupus and Refractory Anemia

**DOI:** 10.7759/cureus.76134

**Published:** 2024-12-21

**Authors:** Isabel Bessa, Elisabete Ribeiro, Teresa Frazão, Anabela De Carvalho, Carlos Fernandes

**Affiliations:** 1 Internal Medicine, Hospital da Senhora da Oliveira, Guimarães, PRT

**Keywords:** hemolytic anemia, lupus, lupus nephritis, nephrotic syndrome, thrombocytopenia

## Abstract

Systemic lupus erythematosus (SLE) is a multisystemic connective tissue disease with a wide range of clinical and laboratory manifestations. The diagnosis of SLE is often challenging due to the great variability in its presentation, and treatment should be individualized according to the patient’s manifestations and affected organs.

We present the clinical case of a 25-year-old female who developed SLE with severe hematological and renal involvement as first manifestations, including hemolytic anemia, thrombocytopenia, and nephrotic syndrome. Diagnosis of SLE was confirmed after positive high titers of ANA and anti-dsDNA antibodies. A kidney biopsy confirmed lupus nephritis class II.

Despite successive treatment with corticosteroids, hydroxychloroquine, intravenous immunoglobulin, and mycophenolate mofetil, there was no hematological improvement, and rituximab was administered, resulting in partial response, with resolution of thrombocytopenia and stabilization of kidney function. However, anemia remained refractory, and subsequent tests indicated a non-hemolytic cause, most likely due to iatrogenic bone marrow suppression. After discontinuing potentially myelotoxic agents, the patient's hemoglobin levels normalized.

This case highlights the intricate challenges associated with managing SLE. It underscores the critical importance of ongoing reassessment of therapeutic strategies, particularly in situations where the treatment response is inadequate. This approach enables the optimization of interventions to improve clinical outcomes and address the unique needs of each patient.

## Introduction

Systemic lupus erythematosus (SLE) is a multisystemic connective tissue disease with a wide range of clinical and laboratory manifestations. It is one of the most frequent autoimmune disorders in women of childbearing age, with an estimated global incidence of 5.14 per 100,000 persons a year [1]. While the etiology of SLE is thought to be multifactorial (linked with environmental, genetic, and hormonal factors), the disease is characterized by the production of autoantibodies, which leads to immune complex deposition, inflammation, and, eventually, organ damage [2]. The most common manifestations are articular, cutaneous, hematologic, renal, cerebral, pulmonary, and vascular, but it can affect any organ [3].

The diagnosis of SLE is often challenging due to the multiplicity of presentation. To help classify SLE cases, the European Alliance of Associations for Rheumatology (EULAR) developed the 2019 EULAR/ACR classification criteria for SLE that includes one entry criteria (a positive ANA titer), seven clinical (constitutional, hematologic, neuropsychiatric, mucocutaneous, serosal, musculoskeletal, renal) and three immunological (antiphospholipid antibodies, complement proteins, SLE-specific antibodies) domains [4]. Each parameter is assigned a weighted score based on its severity and clinical importance. It classifies as SLE if it scores more than 10.

Treatment strategies for SLE should be carefully tailored to each patient, considering their specific clinical manifestations, the severity and extent of organ damage, and any coexisting conditions or comorbidities. The goal is to achieve disease remission or maintain a state of low disease activity, preventing organ damage or its progression, as this significantly impacts long-term health outcomes. By prioritizing these goals, treatment should not only seek to improve physical health but also aim to enhance the patient's overall quality of life.

## Case presentation

A 25-year-old woman presented to the emergency room after a month of diarrhea (two to four bowel movements with loose, watery stools daily), loss of appetite, asthenia, lethargy, and weight loss (5kg). On examination, the patient’s tympanic temperature was 37.8ºC, and blood pressure was 166/120 mmHg. She presented with periorbital and lower limb edema and palpable lymph nodes, 1-2 cm in diameter, in the submandibular, cervical, and inguinal regions.

**Figure 1:**
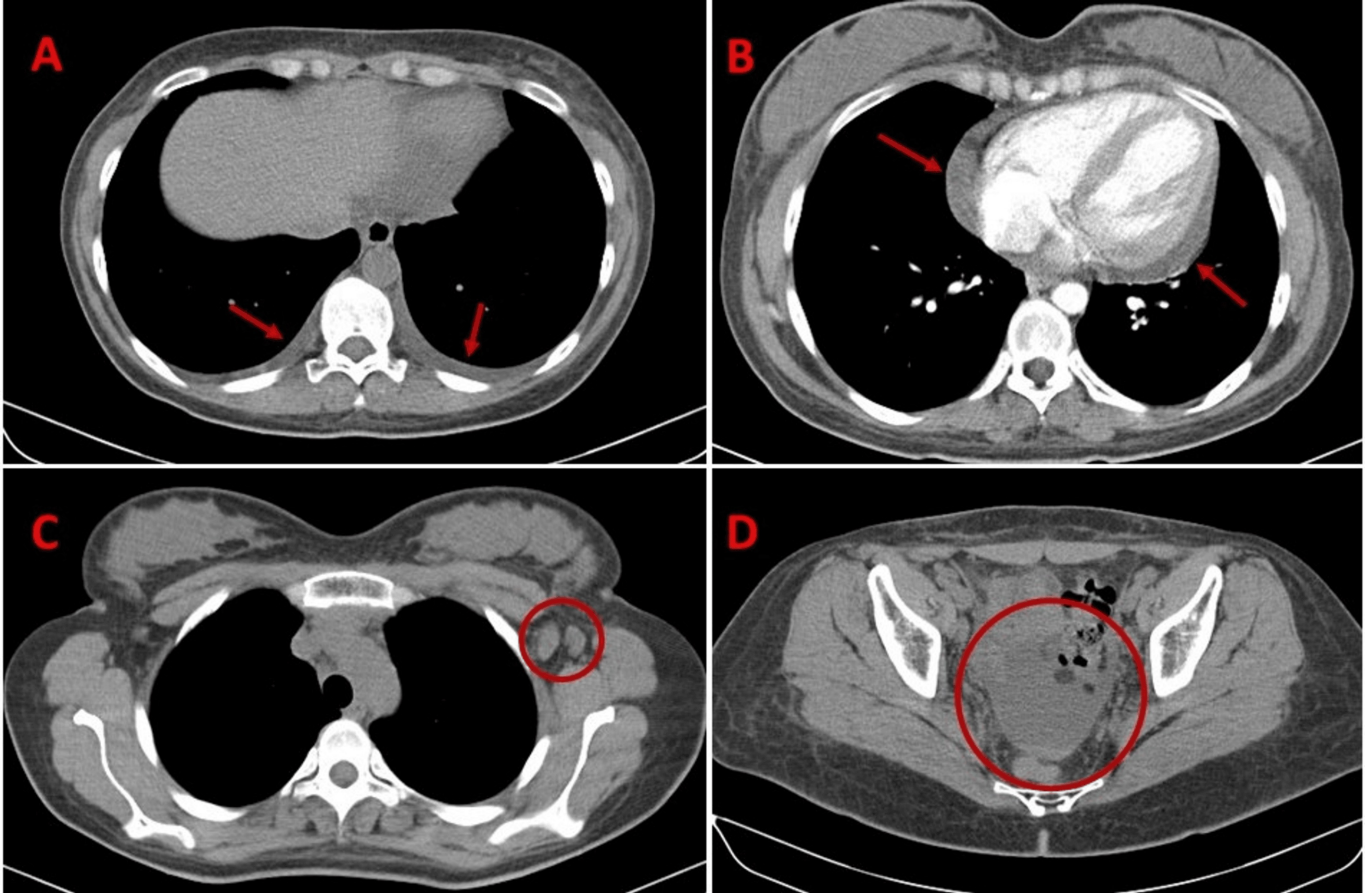
CT-scan findings on admission. These images show the CT-scan findings on admission.  A: shows the pleural effusion (marked by the arrows); B: shows the pericardial effusion (marked by the arrows); C: shows the axillary lymph nodes (highlighted by the circle); D: shows small volume ascites (highlighted by the circle).

Blood tests showed anemia (hemoglobin: 8 g/dL; normal range 12.0-15.5 g/dL), thrombocytopenia (platelet count 25,000 μL; normal range 150,000-450,000 platelets/μL), and kidney injury (creatinine: 1.66 mg/dL; normal range 0.7-1.1 mg/dL). A Direct Coombs test was positive, warm-reactive antibodies of the IgG isotype were detected, and haptoglobin was low. Urinalysis showed proteinuria and erythrocyturia. A CT scan was performed and showed axillary lymph nodes with 2 cm of diameter, a small bilateral pleural and pericardial effusion, and small volume ascites (as shown in Figure 1). No other significant abnormalities were found. The patient was admitted for further study, and 1 mg/kg/day of oral prednisolone was started.

The urine protein/creatinine ratio showed nephrotic proteinuria; Triglycerides were high, and albumin was low. A kidney biopsy was not performed at this time because of severe thrombocytopenia. Complement C3 and C4 were low, Anti-dsDNA were high, and ANA screening was positive, with a homogenous nuclear pattern (typically associated with SLE). Antiphospholipid antibodies were negative. 

An inguinal lymph node was excised, revealing features of nonspecific reactive lymphadenitis. A myelogram was performed to exclude lymphoma or leukemia, but no relevant alterations were found. A diagnosis of SLE, with hematological (hemolytic anemia and thrombocytopenia) and renal involvement, was established. At presentation, the SLE Disease Activity Index 2000 (SLEDAI-2K) was 21 points, indicating very high disease activity (Table 1).

**Table 1:** Systemic lupus erythematosus disease activity index 2000 (SLEDAI-2K) at presentation. SLEDAI-2K stratifies the severity of SLE and is used to monitor changes in disease activity over time. It evaluates 24 clinical and laboratorial parameters; each parameter is assigned a weighted score based on its severity and clinical importance. Score interpretation: 0 points means no activity; 1-5 mild disease activity; 6-12 moderate disease activity; >12 high disease activity.

Descriptor	Points
Recent onset seizure	0
Psychosis	0
Organic brain syndrome	0
Visual disturbance	0
New onset sensory or motor neuropathy	0
Lupus headache	0
New onset stroke	0
Vasculitis	0
Arthritis	0
Myositis	0
Heme-granular or RBC urinary casts	+4
Hematuria	+4
Proteinuria	+4
Pyuria	0
Inflammatory-type rash	0
Alopecia	0
Oral or nasal mucosal ulcers	0
Pleuritic chest pain with pleural rub/effusion or pleural thickening	+2
Pericarditis	0
Low complement	+2
High DNA binding	+2
Temp >38°C	+1
Platelets <100 x10³/uL	+1
WBC <3 x10³/uL	+1
Total	21

Hydroxychloroquine was started when the SLE diagnosis was established (on day seven of hospitalization). Trimethoprim-sulfamethoxazole (TMP-SMX), calcium carbonate, and cholecalciferol were added as prophylactic therapy.

Despite treatment, hemoglobin reached 5 g/dL, and a blood transfusion was needed. A three-day course of intravenous immunoglobulin was added on without response. Mycophenolate mofetil (1g every 8 hours) was then added on. After a week, the diarrhea resolved, but hemoglobin continued to drop (3.9 g/dL), platelets were still at 33,000, and serum creatinine was raised to 1.99 mg/dL. Rituximab was started on day 24 of hospitalization (1g IV administered twice, two weeks apart).

After two doses of rituximab, thrombocytopenia and leucopenia resolved, and kidney function was getting better (Table 2). Anti-ds DNA titers decreased. There was also a resolution of the pleural and peritoneal effusion. But anemia did not improve (hemoglobin 3.4-6.6 g/dL). 

**Table 2:** Evolution of the laboratory tests throughout hospitalization. ANA: antinuclear antibodies.

Analysis (normal value)/timing	Day 1	Day 7	Day 17	Day 19	Day 60	Day 74	Last appointment
Hemoglobin (12.0-16.0 g/dL)	8.0	6.2	5.2	3.9	5.3	8.8	14.2
Leukocytes (4.8-10.8x10³/uL)	2.9	4.4	4.6	3.9	6.0	5.8	4.2
Platelets (150-350x10³/uL)	25	59	22	47	162	139	217
Creatinine (0.72-1.25 mg/dL)	1.66	1.3	1.6	1.91	1.23	0.86	0.78
Albumin (3.4-5.0 g/dL)	2.4	2.3	2.0	2.5	3.6	-	4.8
Urinary proteinuria (g/24h)	5.0	-	-	-	1.1	0.8	0.4
Triglycerides (<150 mg/dL)	214	-	-	-	-	-	-
Haptoglobin (16-200 mg/dL)	<7.8	-	-	-	-	122	-
Anti-DNAds (<27 UI/ml)	-	153	-	-	9.8	9.4	17.7
Complement C3 (82-170 mg/dL)	-	27.5	-	-	-	74.5	93.4
Complement C4 (12-36 mg/dL)	-	0.7	-	-	-	5.7	14
ANA (<1/160)	-	1/320	-	-	-	1/640	-

Considering the refractoriness of the anemia, the patient was transferred (on day 60 of hospitalization) to another hospital center to evaluate the possible use of plasmapheresis. Before considering this technique, the nature of the anemia was questioned, and a Direct Coombs test was repeated. It was negative, and the haptoglobin was normal.

Iatrogenic myelosuppression was considered the most likely cause of the anemia. Mycophenolate mofetil, hydroxychloroquine, and TMP-SMX were discontinued, taking into consideration its potential myelotoxic effect. In a few days, red blood cell count began to rise. After 10 days, hydroxychloroquine was reintroduced, and atovaquone replaced TMP-SMX as a prophylaxis against *Pneumocystis jirovecii*.

The patient was discharged after 74 days of hospitalization. She was clinically better, showing improvement of the peripheral edema, with controlled blood pressure under indapamide 2.5 mg and losartan 100 mg. The cytopenias and renal function were normalized (see Table 2 to check the analytical evolution throughout the hospitalization). The patient was maintained on hydroxychloroquine and prednisolone. A week after discharge, the patient was readmitted to our hospital to undergo elective kidney biopsy. A lupus nephritis class II was diagnosed.

In the follow-up consultations, mycophenolate mofetil was reintroduced, and prednisolone was suspended after 11 months. Currently, after two years, the disease is in remission (SLEDAI-2K score of 0). The current therapy is 200 mg/day of hydroxychloroquine and mycophenolate 1000 bid.

## Discussion

This case illustrates the complexity of SLE in a young female patient. The initial presentation included constitutional symptoms such as weight loss and asthenia, diarrhea (a less common primary manifestation of SLE), alongside edema and lymphadenopathy. Laboratory findings revealed severe hemolytic anemia and thrombocytopenia. The positive ANA, low complement levels, along with elevated anti-dsDNA antibodies supported the diagnosis of SLE. A SLEDAI-2K score of 21 points indicated very high disease activity, emphasizing the severity of the patient's presentation. Lupus nephritis was assumed, based on renal involvement, as nephrotic syndrome, even though severe thrombocytopenia precluded an early kidney biopsy.

Treatment included corticosteroids, hydroxychloroquine, IV immunoglobulin, and later mycophenolate mofetil, which are standard therapies for managing SLE flares. Despite these interventions, the patient's anemia and thrombocytopenia persisted, indicating a refractory state of disease. Rituximab and cyclophosphamide could be used as 1st line treatment in severe SLE [5]. Cyclophosphamide can cause direct damage to the ovarian follicles, which can result in premature ovarian failure or early menopause, and it is also teratogenic [6]. Since our patient was a young woman, cyclophosphamide was not our first choice. Rituximab was initiated after a lack of response to conventional treatments. Despite improvement in thrombocytopenia and renal function, the persistence of severe anemia raised concerns.

Plasma exchange is used in many antibody-mediated disorders, such as myasthenia gravis, vasculitis, antiphospholipid syndrome, and glomerular disorders (i.e., ANCA-associated vasculitis, focal segmental glomerulosclerosis or anti-GBM disease). It might also be an option for SLE patients who fail to use any of the aforementioned therapies [7,8]. 

Reevaluating the etiology of anemia was essential and ultimately prevented the need for this complex procedure. All other parameters were improving except for anemia. Although hemolysis was no longer occurring (as confirmed by a negative Direct Coombs test and normal haptoglobin levels), hemoglobin levels continued to decline and only began to improve a few days after discontinuing the potentially myelotoxic drugs. This observation supports the hypothesis of myelotoxicity from immunosuppressive therapies. All immunosuppressive drugs can potentially cause myelosuppression. Although azathioprine and cyclophosphamide are most frequently associated with this risk, mycophenolate can also lead to cytopenias [9,10]. TMP-SMX can also induce myelosuppression, especially in the setting of underlying immunosuppression (as in this case) [11,12]. Even though TMP-SMX is the most commonly used drug for prophylaxis in autoimmune inflammatory diseases, Atovaquone is an alternative medication for preventing *Pneumocystis jirovecii* pneumonia. It appears to be equally effective and possibly safer than TMP-SMX in SLE [13].

Future research should focus on developing and optimizing alternative therapies for patients who do not respond to standard treatments. New biologics, non-teratogenic and less toxic immunosuppressants that are effective in severe cases could be valuable for young patients, especially those of childbearing age. It should also focus on evaluating the role of plasma exchange in SLE. More controlled studies are needed to determine when plasmapheresis is beneficial and to develop guidelines for its use in refractory cases. Non-invasive biomarkers to detect and classify lupus nephritis could help manage cases with contraindications to biopsy. Reliable biomarkers could improve the diagnosis and monitoring of renal involvement without invasive procedures. By addressing these areas, future research could improve the management of severe, refractory SLE and reduce the burden of treatment-related side effects, ultimately enhancing patient outcomes and quality of life.

## Conclusions

This case underscores the complexity of diagnosing and managing severe, refractory SLE. The presentation of atypical symptoms, severe hematologic abnormalities, and renal involvement required a nuanced diagnostic approach and individualized therapeutic decisions. The choice of treatment balanced the need for disease control with the minimization of treatment-related risks, such as myelotoxicity and long-term reproductive consequences. Long-term follow-up and careful adjustment of therapy are essential to achieve remission and manage the side effects of immunosuppressive treatments.
